# Serum Metabolic Profiling of Oocyst-Induced *Toxoplasma gondii* Acute and Chronic Infections in Mice Using Mass-Spectrometry

**DOI:** 10.3389/fmicb.2017.02612

**Published:** 2018-01-04

**Authors:** Chun-Xue Zhou, Wei Cong, Xiao-Qing Chen, Shen-Yi He, Hany M. Elsheikha, Xing-Quan Zhu

**Affiliations:** ^1^State Key Laboratory of Veterinary Etiological Biology, Key Laboratory of Veterinary Parasitology of Gansu Province, Lanzhou Veterinary Research Institute, Chinese Academy of Agricultural Sciences, Lanzhou, China; ^2^Department of Parasitology, Shandong University School of Basic Medicine, Jinan, China; ^3^Department of Prevention and Treatment of Animal Diseases, College of Marine Science, Shandong University (Weihai), Weihai, China; ^4^Department of Microbiology and Immunology, College of Animal Science and Technology, Jilin Agricultural University, Changchun, China; ^5^Faculty of Medicine and Health Sciences, School of Veterinary Medicine and Science, University of Nottingham, Loughborough, United Kingdom

**Keywords:** *Toxoplasma gondii*, oocyst, LC-MS/MS, metabolomics, mouse model

## Abstract

*Toxoplasma gondii* is an obligate intracellular parasite causing severe diseases in immunocompromised individuals and congenitally infected neonates, such as encephalitis and chorioretinitis. This study aimed to determine whether serum metabolic profiling can (i) identify metabolites associated with oocyst-induced *T. gondii* infection and (ii) detect systemic metabolic differences between *T. gondii*-infected mice and controls. We performed the first global metabolomics analysis of mice serum challenged with 100 sporulated *T. gondii* Pru oocysts (Genotype II). Sera from acutely infected mice (11 days post-infection, dpi), chronically infected mice (33 dpi) and control mice were collected and analyzed using LC-MS/MS platform. Following False Discovery Rate filtering, we identified 3871 and 2825 ions in ESI+ or ESI− mode, respectively. Principal Component Analysis (PCA) and Partial Least Squares Discriminant Analysis (PLS-DA) identified metabolomic profiles that clearly differentiated *T. gondii*-infected and -uninfected serum samples. Acute infection significantly influenced the serum metabolome. Our results identified common and uniquely perturbed metabolites and pathways. Acutely infected mice showed perturbations in metabolites associated with glycerophospholipid metabolism, biosynthesis of amino acid, and tyrosine metabolism. These findings demonstrated that acute *T. gondii* infection induces a global perturbation of mice serum metabolome, providing new insights into the mechanisms underlying systemic metabolic changes during early stage of *T. gondii* infection.

## Introduction

*Toxoplasma gondii* is an important intracellular protozoan parasite that is able to cause encephalitis in immuno-compromised individuals, chorioretinitis in immunocompetent patients and abortion in pregnant women (Montoya and Remington, 1996; Yarovinsky, 2014). Reproductive disorders, with teratogenic effects, abortions, and stillbirths are the main economic impact of *T. gondii* on livestock. Among the food animals, *T. gondii* infections are more prevalent in pigs, sheep, and goats than in cattle (Lopes et al., 2013). The life cycle of this parasite involves asexual phase in intermediate vertebrate host and sexual phase occurs exclusively in the intestinal epithelium of the definitive felid host (Sullivan and Jeffers, 2012). Sexual reproduction results in the formation and shedding of unsporulated (uninfective) oocysts, which become infectious/sporulated within a few days (Dubey et al., 2011). Sporulated oocysts can survive for 18 months in soil (Frenkel et al., 1975), remain infectious for mice when stored at 4°C for 24 months in seawater (Lindsay and Dubey, 2009) and have a remarkable ability to survive under adverse climatic conditions (Dumètre et al., 2013). Although ingestion of *T. gondii* cysts-contaminated meat has been the primary source of human infection, improvement in animal management and husbandry practice has significantly reduced *T. gondii* prevalence in meat (Jones and Dubey, 2012). In the meantime, waterborne outbreaks linked to drinking *T. gondii* oocysts-contaminated water have been increasingly reported, posing a public health threat (Boyer et al., 2011).

As an obligatory intracellular pathogen, *T. gondii* reproduces by co-opting the resources of its host cells and inevitably perturb the host cells at the transcription, protein and metabolite levels. The complexity of host-*T. gondii* interactions requires a system level understanding of the entire hierarchy of biological interactions and dynamics. Therefore, several “omics” techniques, including transcriptomics (Pittman et al., 2014), proteomics (Zhou et al., 2013), and metabolomics (Zhou et al., 2015, 2016b) have been developed and employed to interrogate this intricate relationship between *T. gondii* and its host. Among these “omics” platforms, metabolomics offers an advantage when trying to decipher disease pathogenesis because metabolites are more proximal to a phenotype than transcriptional or protein information and thus can reflect the physiological status of the human body, more than other omics-based approaches. The ability to capture the global chemical changes makes metabolomics a powerful bioanalytical tool to elucidate sites of perturbations in biochemical pathways in any biological system. We have previously utilized metabolomics to identify biomarkers in the serum and brain of mice infected with *T. gondii* cysts (Zhou et al., 2015, 2016b). Our recent studies enabled the assessment of a broad range of endogenous metabolites and identified perturbed biochemical pathways in response to infection. However, it is still not well-understood which metabolic pathways *T. gondii* uses for growth and multiplication within the host. Also, despite the public health importance and the key role that oocysts play in the life cycle of *T. gondii* metabolic signature associated with *T. gondii* oocyst infection is unknown.

Although *T. gondii* is an obligate intracellular parasite, host serum offers an important window for understanding host/parasite biology during infection. Moreover, metabolomic analysis of serum samples provides a powerful tool for phenotypic biology and clinical biomarker discovery, when compared to *in vitro* studies of parasite biology. Aiming to gain better insights into *T. gondii* pathogenesis and to identify new biomarkers with potential diagnostic or therapeutic values we have investigated the serum metabolic profile of mice infected with *T. gondii* sporulated oocysts using non-targeted LC-MS/MS approach. We successfully identified alterations in several metabolites and specific metabolic pathways, such as amino acid and energetic metabolism, in mice serum during acute and chronic phases of *T. gondii* infection.

## Materials and methods

### Ethical approval

The maintenance and care of experimental animals was carried out in strict accordance with the Animal Ethics Procedures and Guidelines of the People's Republic of China. All animal experiments used in this study were approved by Animal Ethics Committee of Lanzhou Veterinary Research Institute, Chinese Academy of Agricultural Sciences (Permit No. LVRIAEC2014-002). All efforts have been made to alleviate suffering and minimize the number of animals used in the study.

### Oocyst production and purification

The majority of human toxoplasmosis cases were associated with strains of a genotype II (Howe and Sibley, 1995). Oocysts of *T. gondii* Pru strain (type II) were obtained and purified according to a previously described method (Zhou et al., 2016a). Briefly, tissue cysts of *T. gondii* Pru strain were obtained from infected Kunming mice. After verification of the tissue cyst status by histological examination, a 10-week-old specific-pathogen-free kitten was fed with approximately 100 freshly prepared cysts (Zhou et al., 2016a). Feces of the infected kitten were examined daily for the detection of the oocysts. Oocysts were isolated from feces using a discontinuous cesium chloride gradient method as previously described (Staggs et al., 2009). To induce sporulation, the purified oocysts were incubated in 2% H_2_SO_4_ and aerated on a shaker for 7 days at ambient temperature. Sporulated oocysts were examined with a light microscope (Olympus, Japan).Finally, the oocysts were washed twice in 0.85% saline and were suspended in 2% H_2_SO_4_ and stored in aliquots that were maintained at 4°C.

### Mouse infection

The mice used in this study were female, 6 to 8-week-old, of the BALB/c strain, and were obtained from Lanzhou University, China and were maintained under specific-pathogen-free conditions in a standard experimental facility at Lanzhou Veterinary Research Institute, Chinese Academy of Agricultural Sciences. Mice received sterilized food and water *ad libitum*. Twelve mice were infected orally with ~100 freshly prepared sporulated oocysts via oral gavage with 100 μL saline solution using 1^−ml^ tuberculin syringes and feeding needles. Six mock-infected (control) mice received 100 μL saline alone. All mice were monitored daily for mortality and morbidity throughout the course of infection. At 11 and 33 days post-infection (dpi) with *T. gondii* oocysts six mice were sacrificed. The infection in each mouse was verified by PCR analysis of DNA extracted from mice tissues, brain, blood, liver, spleen, lung, small intestine, and kidney, targeting the B1 region of *T. gondii* as previously described (Hill et al., 2006). Also, mice spleen, kidney and liver were collected and fixed in 10% buffered formalin (pH 7.2) for 1 week followed by dehydration through a graded series of alcohol to xylol and embedded in paraffin wax. Sections of 5 μm thick from paraffin wax blocks were cut and stained with hematoxylin and eosin (H & E) for histopathological analysis.

### Serum metabolite extraction

The blood samples from acutely infected (11 dpi), chronically infected (33 dpi) and control mice groups were prepared as previously described (Wikoff et al., 2009). Briefly, blood samples were obtained by retro-orbital bleed into Eppendorf tubes and were allowed to clot for 3 h followed by centrifugation at 3,000 g for 10 min at 4°C. Sera were frozen immediately in liquid nitrogen and stored at −80°C until use for metabolite extraction. Initially, frozen serum samples were placed at −20°C for 30 min and then thawed at 4°C. Serum sample aliquots (40 μL) were treated with 120 μL methanol (3:1 vol/vol) and mixed for 1 min. The mixture was kept at −20°C for 30 min, followed by centrifugation at 4,000 g for 20 min at 4°C. The supernatant (20 μL) was mixed with 180 μL methanol before liquid chromatography separation.

### UPLC-MS/MS analysis

Liquid chromatography was performed on a 2777C UPLC system (Waters, UK). The separation of all samples was performed on an ACQUITY UPLC BEH C18 column (Waters, U.K.) (100 × 2.1 mm, 1.7 μm). A gradient elution program was run for chromatographic separation with mobile phase A (water) and mobile phase B (acetonitrile) as follows: 0~2 min, 100%A-100%A; 2~11 min, 100%A-0%A; 11~13 min, 0%A-0%A; 13~15 min, 0% A-100%A. The injection volume was 10 μL and the flow rate was set as 0.4 mL/min. A SYNAPT G2 XS QTOF (Waters, UK) equipped with an electrospray ionization (ESI) source was used for mass spectrometric detection. Sample analysis was performed in both positive and negative ion modes. The operating parameters were as follows: capillary, 3kV(ESI+) or 0.33 kV(ESI−); sampling cone: 40V; source temperature: 110°C (ESI+) or 120°C (ESI−); desolvation temperature: 550°C (ESI+) or 400°C (ESI−); desolvation gas, 800 (L/h) (ESI+) or 500 (L/h) (ESI−); cone gas, 55 (L/h) (ESI+) or 60 (ESI−); source offset: 80; TOF acquisition mode: sensitivity (ESI+) or sensitivity (ESI−); acquisition method, continuum MS^E^; TOF mass range: 50–1,200 Da; scan time: 0.2 s; collision energy function 2: trap CE ramp 20 to 40 eV. Quality control (QC) samples were used in order to assess the reproducibility and reliability of the LC-MS/MS system. QC samples were prepared by mixing equal volumes (20 μL) from each serum sample as they were aliquoted for analysis. This “pooled” sample was used to provide a “mean” profile representing all analytes encountered during the analysis. The pooled “QC” sample was run 10 times at the beginning of the analysis and then prior to and after each batch of 10 serum samples to ensure system equilibration.

### Data processing and analysis

Raw data files were uploaded into Progenesis QI 2.1 software (Waters Corp., Manchester, UK), which was used to perform untargeted peak detection, peak alignment, peak grouping, normalization, and integration on each full data set (experimental study samples and QC samples). Then, the data matrix was mean-centered and Pareto-scaled prior to multivariate analysis (MVA) using Principal Component Analysis (PCA) and Partial Least Squares Discriminant Analysis (PLS-DA). PCA and PLS-DA were performed to discriminate infected from control mice. The quality of the models was evaluated with the relevant *R*^2^ and *Q*^2^ as discussed elsewhere (Lee et al., 2003). The non-parametric univariate method (two-tailed Wilcoxon rank-sum test) was used to discover the significantly altered metabolites among the infected and healthy control mice. The results were corrected by false discovery rate (FDR) to ensure that metabolite peaks were reproducibly detected. The differential metabolites were selected when the statistically significant threshold of variable importance in the projection (VIP) values obtained from the PLS-DA model were larger than 1.0 (Emond et al., 2013). Statistically significant differences were tested using Student's *t*-test, and the corrected *P*-values (*q*-value) <0.05 was deemed as statistically significant. Log_2_ fold change (FC) was used to show how the selected differential metabolites varied between the mice groups. Serum metabolites passing the VIP threshold (VIP > 1) and fold change ≥1.2 or ≤0.8 were considered as significantly different between the mice groups (Feng et al., 2016).

Heatmaps were used to depict the relatively disturbed and unbalanced metabolic signature among *T. godnii*-infected mice compared to control mice. Heatmaps were generated using the MultiExperiment Viewer (MeV) v. 4.9 software (http://mev.tm4.org/) based on the abundance of the differentially expressed metabolite data (log2-scaled) (Halama et al., 2015). The online databases of Human Metabolome Database (HMDB; http://www.hmdb.ca) and Kyoto Encyclopedia of Genes and Genomes (KEGG; http://www.genome.jp/kegg) were used to check and confirm the putative identity of the differentially expressed metabolites by matching the exact molecular mass data (m/z) of samples with those from the database (Wishart et al., 2013; Du et al., 2014). The candidate metabolites were confirmed by MS/MS scans for the characteristic ions and fragmentation patterns of the compound. We performed pathway analysis of the differentially expressed metabolites detected in both ion modes using MetaboAnalyst 3.0 (Xia et al., 2015). The identified pathways influenced by acute infection compared to controls are presented according to *p*-values from the pathway enrichment analysis (y-axis) and pathway impact values from pathway topology analysis (x-axis), with the most impacted pathways colored in red color. To gain further insight into the underlying biological mechanisms associated with infection, we performed the impact value and –log (*P*) along with metabolic pathway analysis (MetPA), the *P*-value represented the enrichment of certain metabolites in a pathway (*P* ≤ 0.05 is indicative of significant enrichment).

## Results

### Characteristics of infected mice

Purified sporulated oocysts used in this study are shown in Figure S1. In infected mice, signs of illness started at 5–7 dpi and peaked at 11 dpi when mice developed severe splenomegaly and other signs consistent with acute infection, including anorexia, weight loss, edema, and messy hair. As infection progressed animals slowly started to recover and by 33 dpi infected mice had restored their physical status and developed chronic infection as indicated by the observation of *T. gondii* tissue cysts in homogenized brains at 33 dpi (Figure S2). Infection was confirmed via the detection of *T. gondii* B1 gene in various mouse tissues of all infected mice (Table S1). Mice infected with *T. gondii* exhibited a disruption of their splenic architecture. Histopathological changes on day 11 post-infection included a structural breakdown, reduced white pulp and increase in the number of megakaryocytes (Figure S3A). On day 33 post infection, the red and white pulp were observed. However, the white pulp showed mild hyperplasia and some lymphoid follicles tended to coalesce, and the red pulp was non-reactive (Figure S3B). As expected, mice in the control group appeared clinically normal during the experiment, were PCR-negative for *T. gondii* infection and did not show any histopathological abnormalities (Figure S3C).

### Serum metabolic profiles

**Figure 1:**
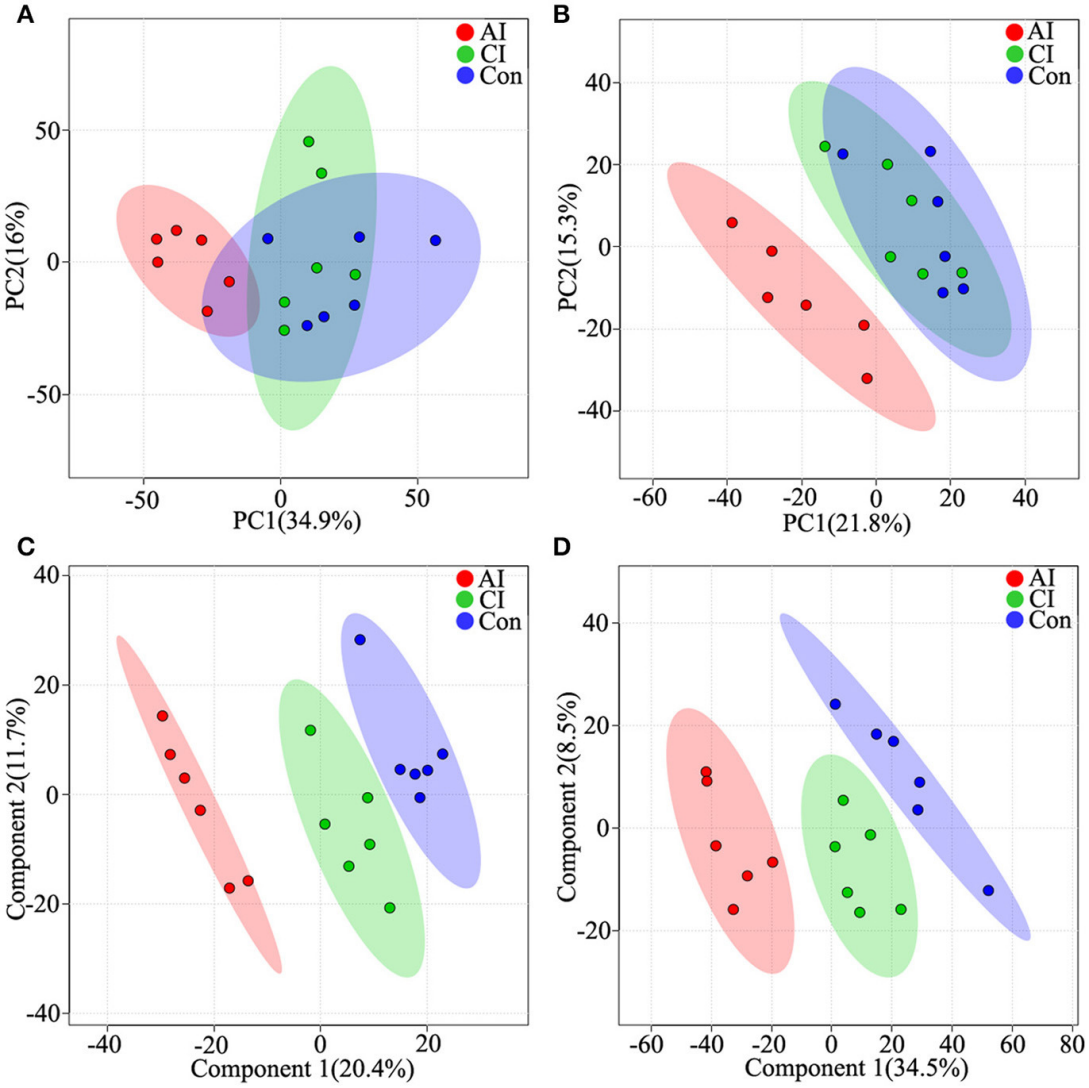
Differential metabolic profiles of mice serum during the course of *T. gondii* infection. Principal component analysis (PCA) score scatter plots of metabolites obtained from LC-QTOF-MS/MS fingerprints in ESI+ **(A)** and ESI− mode **(B)**. Partial least-squares discriminant analysis (PLS-DA) separating metabolites of the three groups in ESI+ mode **(C)** and ESI− mode **(D)**. Acutely infected group (AI, red), chronically infected group (CI, green) and control group (Con, blue). Ellipses enclose the 95% confidence intervals estimated by the sample means and covariances of each group.

To access the capability of the LC-MS/MS based-metabolomics approach used in this study we analyzed all total ion chromatograms of QC samples, which exhibited a stable retention time without obvious peaks' drifts. There were 4,583 and 3,388 ions identified in each sample profile in ESI+ and ESI− mode, respectively. After removing low-quality ions [relative standard deviation (RSD)>30%], 3,871 and 2,825 ions in each sample were identified in ESI+ or ESI− mode, respectively. To assess the reproducibility of our dataset, QC samples were measured during the entire experiment. PCA among the QC samples and tested samples was performed and the two-dimensional PCA score plots are shown in Figure S4. The results showed that QC samples were clustered closely to each other and were separated from the tested samples, confirming the stability and reproducibility of the LC-MS/MS analysis. Next, we compared the metabolic profiles across the different mice groups by employing two dimensional PCA. Although acutely infected mice were clearly separated from the chronically infected and control mice groups, PCA scores plots under both ion modes did not clearly differentiate chronically infected mice from control group in ESI+ mode (Figure 1A) and ESI− mode (Figure 1B). Further multivariate analysis using cross-validated two-dimensional PLS-DA models was performed, which showed distinct separation of the three mice groups in ESI+ mode (Figure 1C) and ESI− mode (Figure 1D).

### Serum metabolic profiles distinguish between acute and chronic toxoplasmosis

**Figure 2:**
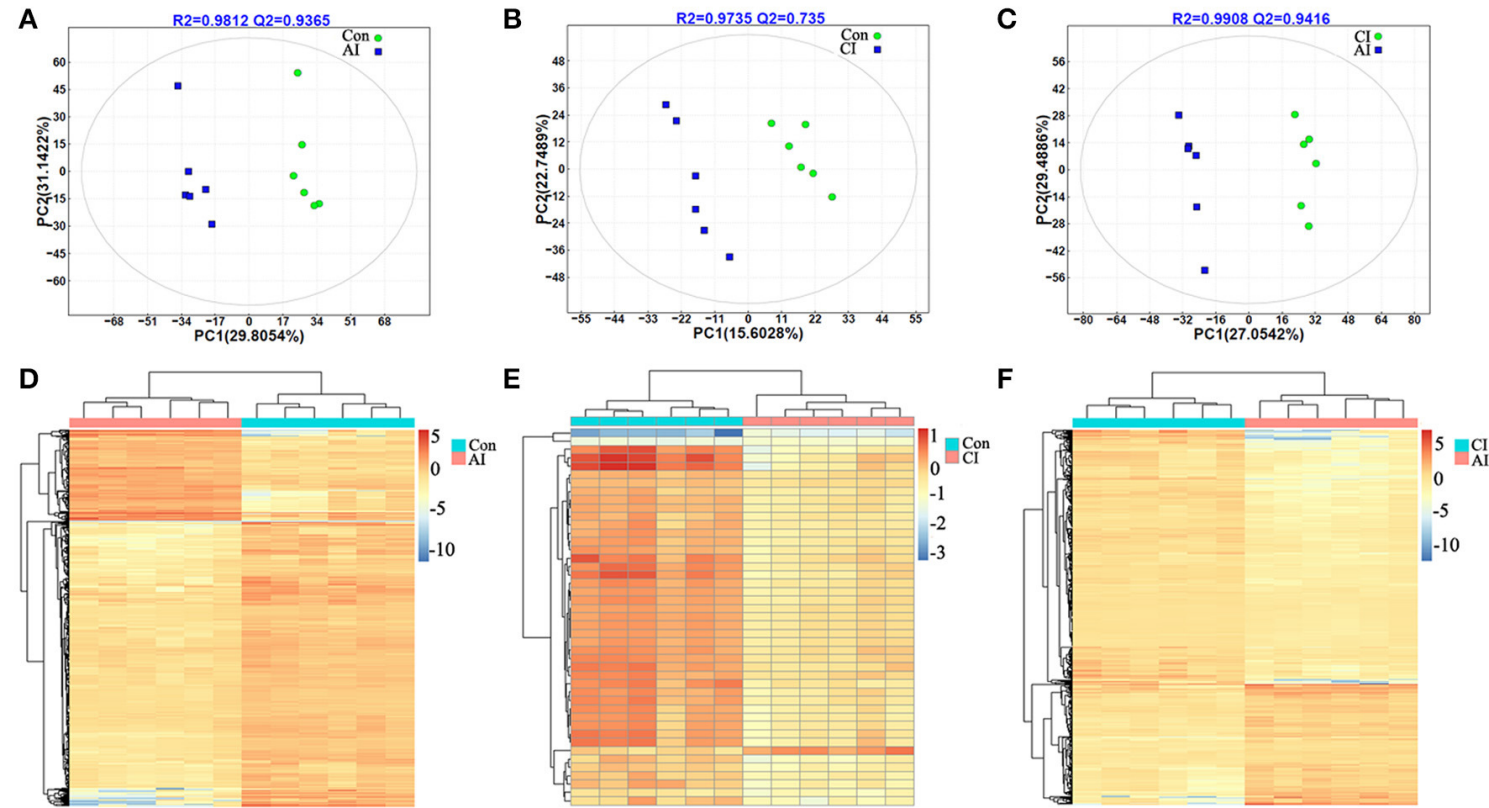
Discrimination between acutely infected, chronically infected and control mice based on ESI+ mode-derived metabolic phenotype of serum. **(A–C)** Partial least squares-discriminate analysis (PLS-DA) score plots of **(A)** acutely infected vs. control, **(B)** chronically infected vs. control and **(C)** acutely infected vs. chronically infected. **(D–F)** Heat maps of the top differential metabolites for **(D)** acutely infected vs. control, **(E)** chronically infected vs. control and **(F)**, acutely infected vs. chronically infected. Sample groups including acutely infected, chronically infected and control, are labeled AI, CI, and Con, respectively.

Score scatter plots for two-dimensional PLS-DA model from both ion modes showed a clear differentiation of acutely infected and control groups. The 12 serum samples are clustered based on the relative MS intensity of these differential metabolite ions detected in ESI+ mode (Figure 2A) and ESI-mode (Figure S5A), which clearly revealed two distinct clusters, corresponding to acutely infected mice and control mice group. This clear separation was also demonstrated in the heatmap based on the 12 serum samples in the ESI+ mode (Figure 2D) and the ESI− mode (Figure S5D).

The scores plots of PLS-DA model clearly discriminated chronically infected mice from control mice as shown in Figure 2B (ESI+) and Figure S5B (ESI−), respectively. Meanwhile, a heatmap constructed based on the significantly differential metabolite ions detected in the ESI+ mode further supported the distinct metabolic features between chronic infection vs. control (Figure 2E). However, in the ESI− mode, there was no any differential metabolite between chronically infected and control mice. So, there is not a heat map for the comparison between these two groups.

Likewise, two-component PLS-DA models showed good discrimination between acute and chronic infection in both ion modes: ESI+ mode (Figure 2C) and ESI− mode (Figure S5C). Interestingly, heatmaps constructed based on differential metabolite ions between acutely and chronically infected mice groups showed a clear clustering (Figure 2F and Figure S5E), which demonstrates the reliability of the PLS-DA models to distinguish between different disease-specific metabolic phenotypes.

### Patterns of differentially abundant metabolites

**Figure 3:**
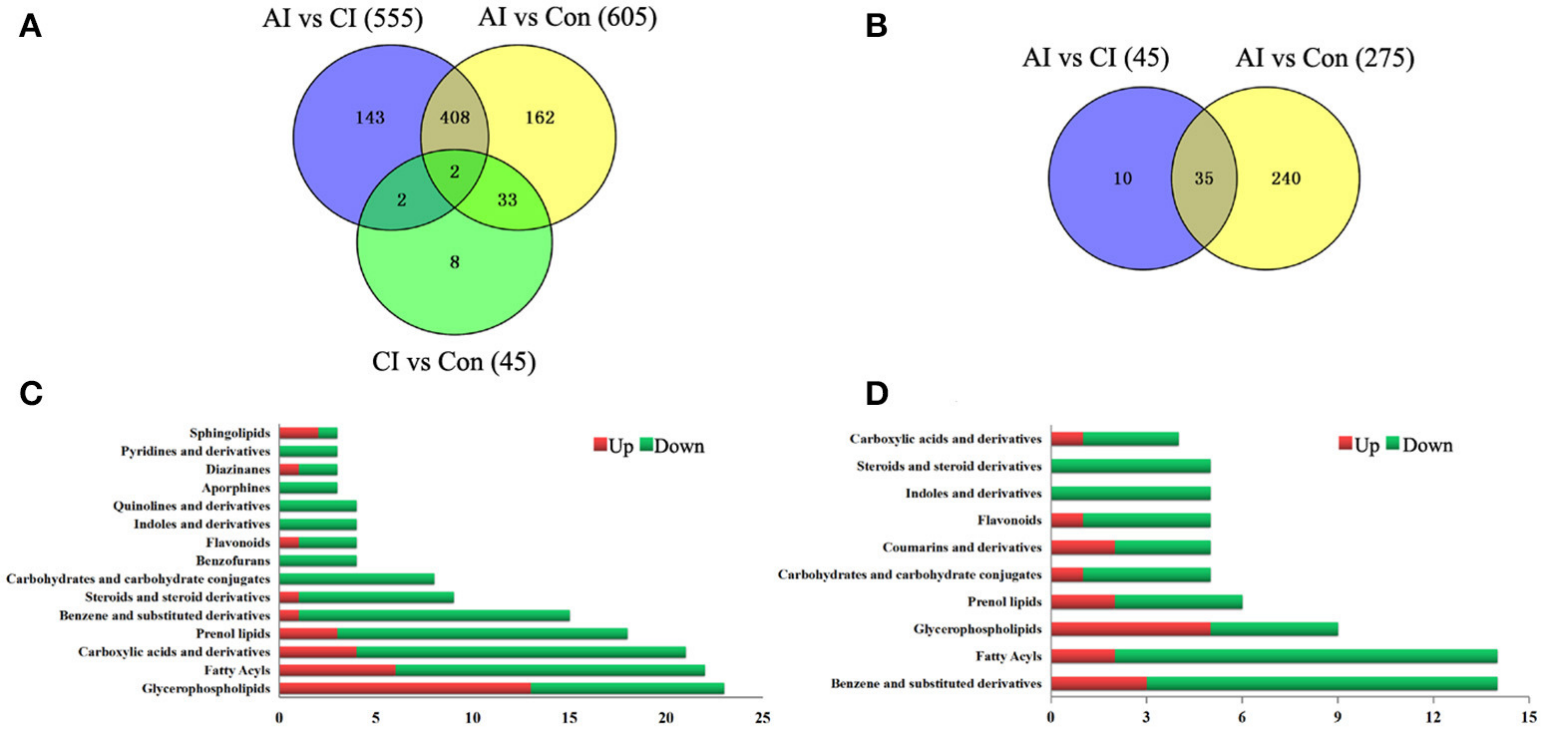
Comparison of metabolomics data across *T. gondii* infection state. Venn diagrams displaying (comparatively) the differentially expressed metabolites. All differentially expressed metabolites are clustered into three comparison groups represented by three circles. The sum of all the figures in one circle represents the number of differentially expressed metabolites in one comparison group (e.g., AI vs. CI). The overlapping parts of different circles represent the number of differentially expressed metabolites shared between these comparison groups. The single-layer part represents the number of metabolites distinctly found in a certain comparison group. **(A,B)** Differential metabolites across comparison groups showing unique and common metabolites in **(A)** ESI+ mode and **(B)** ESI− mode. **(C)** Top 15 enriched metabolite terms of acutely infected vs. control in ESI+ mode. **(D)** Top 10 enriched metabolite terms of chronically infected vs. control in ESI− mode. Red and green metabolites indicate higher and lower concentrations, respectively. The bars on x-axis represent the number of metabolites for the chemical classes mentioned on the y-axis.

As shown in Figure 3A, the serum metabolic profile in infected mice changed significantly from the controls at 11 dpi (ESI+). As the infection progresses the number of differential metabolites decreased, indicating a restoration of the dysregulated metabolic state in a time-dependent manner. Of the 758 differential metabolites, 445 were common across the three comparison pairs. The majority of the differential metabolites (410) were common between AI vs. Con and AI vs. CI. Thirty-five differential metabolites were detected between AI vs. Con and CI vs. Con. Meanwhile, in the ESI− mode, the number of differential metabolites identified between the acute infection group and the controls was the largest (Figure 3B). By searching against mass-based databases, some of these differential monoisotopic ions were putatively identified. As shown in Figure 3C, “glycerophospholipids,” “fatty acyls” and “carboxylic acids and derivatives” were the most prominent categories between acutely infected mice and controls in ESI+ mode. Meanwhile, “benzene and substituted derivatives,” “fatty acyls” and “prenol lipid” were the three most enriched categories between acutely infected and control mice in ESI− mode (Figure 3D).

**Figure 4:**
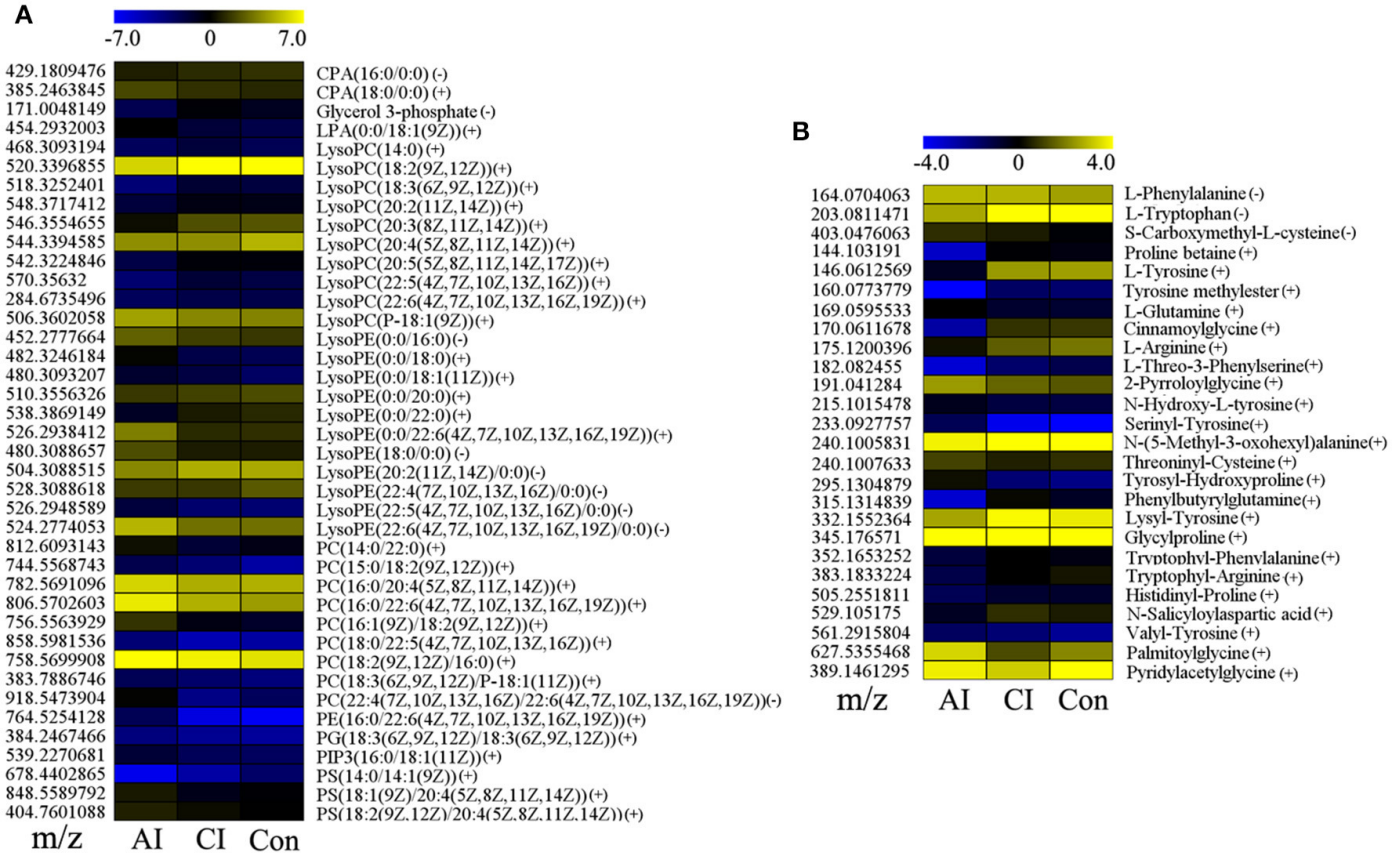
Comparison of glycerophospholipid and amine-containing metabolite levels at different infection stages. **(A)** Heat map of 40 glycerophospholipids during the whole infection process. CPA, cyclic phosphatidic acid; LPA, lysophosphatidic acid; PC, phosphatidylcholines; PE, phosphatidylethanolamines; PG, phosphatidylglycerols; PS, phosphatidylserines. **(B)** Heat map of 26 significant amine-containing metabolites during the whole infection course. The color of each section corresponds to an abundance value of each metabolite calculated by peak area normalization method. Each row represents data for a specific metabolite and each column represents acutely infected (AI), chronically infected (CI) or Control (Con) mouse group. Different colors correspond to the different intensity level of metabolites.

Many perturbed lipid metabolites belonging to glycerophospholipids (Figure 4A). Comparison between chronically infected and control mice showed fatty acyls, benzenes, steroids, and glycerophospholipids as the top 4 most enriched metabolite classes in the ESI+ mode (Figure S6). However, in the ESI− mode no differential metabolites were detected between chronically infected and control mice. Amine-containing metabolites are involved in many biological processes, especially in amino acid and nucleotide metabolic pathways. As shown in Figure 4B, 26 amine-containing metabolites were found perturbed in comparison between different infection groups. As shown in Table 1, we detected 35 metabolites in ESI+ mode that varied at both acute and chronic infection stages. Traumatin, a metabolite of the α-linoleic acid pathway, is the only metabolite that showed up-regulated levels during the whole infection course especially during the acute stage.

**Table 1:** List of dysregulated metabolites identified in ESI+ mode throughout the entire infection process.

**MS (m/z)**	**RT (min)**	**Metabolites**	**AI vs Con**				**CI vs Con**			
			**VIP**	**FC**	***q*****-value**	**CV**	**VIP**	**FC**	***q*****-value**	**CV**
221.0199166	0.604766667	Hydroxyphenyllactic acid	1.83	0.346	1.93E03	↓	2.43	0.472	2.69E02	↓
251.1041128	4.582316667	Traumatin	3.22	27.617	2.94E04	↑	2.61	2.34	2.69E02	↑
280.0935614	0.604766667	Tolmetin	1.78	0.367	3.33E03	↓	2.14	0.56	3.98E02	↓
303.2326059	8.339133333	Yucalexin A16	1.46	0.495	3.33E03	↓	2.10	0.583	1.70E02	↓
305.2485248	8.467716667	Drostanolone	1.53	0.396	1.29E02	↓	2.47	0.468	4.75E02	↓
343.2247794	8.339133333	2-Hydroxy-6-tridecylbenzoic acid	1.47	0.490	4.00E03	↓	2.12	0.578	1.70E2	↓
357.1522945	8.339133333	xi-3-Hydroxy-5-phenylpentanoic acid O-beta-D-Glucopyranoside	1.54	0.422	1.36E02	↓	2.31	0.519	1.70E2	↓
359.0921059	8.7456	1-Hydroxypyrene glucuronide	1.25	0.590	3.21E03	↓	2.07	0.595	2.96E02	↓
359.1541578	8.339133333	N2-Fructopyranosylarginine	1.51	0.454	5.85E03	↓	2.06	0.584	2.69E02	↓
367.1431981	8.339133333	Labetalol	1.44	0.496	5.81E03	↓	2.06	0.594	1.70E2	↓
371.2078265	8.339133333	(3S,7E,9S)-9-Hydroxy-4,7-megastigmadien-3-one 9-glucoside	1.42	0.448	1.66E02	↓	2.23	0.527	1.77E02	↓
377.1637462	8.32485	Trigoforin	1.52	0.436	9.74E03	↓	2.17	0.558	1.70E02	↓
403.1617396	8.32485	Suxibuzone	1.16	0.575	2.22E02	↓	1.90	0.634	2.69E02	↓
406.1726594	8.339133333	18-Carboxy-dinor-LTE4	1.52	0.456	7.16E03	↓	2.22	0.545	1.87E02	↓
408.1700156	8.339133333	Trifluoperazine	1.53	0.441	8.24E03	↓	2.22	0.541	1.90E02	↓
544.3394585	8.7456	LysoPC(20:4(5Z,8Z,11Z,14Z))	1.43	0.487	2.83E03	↓	2.40	0.489	1.70E02	↓
762.177347	8.7456	Valrubicin	1.21	0.603	9.75E03	↓	2.32	0.504	2.69E02	↓

*RT, Retention time; VIP, variable importance for projection; CV, Content variance; AI, CI, and Con denote acute infection group, chronic infection group, and control group; FC, Fold change; q-value, adjusted p-value calculated by the two-tailed Wilcoxon rank-sum tests after false discovery rate correction. A single arrow (↑/↓) indicates increased or decreased values compared to the control group*.

### Altered metabolic pathways and their biological significance

**Figure 5:**
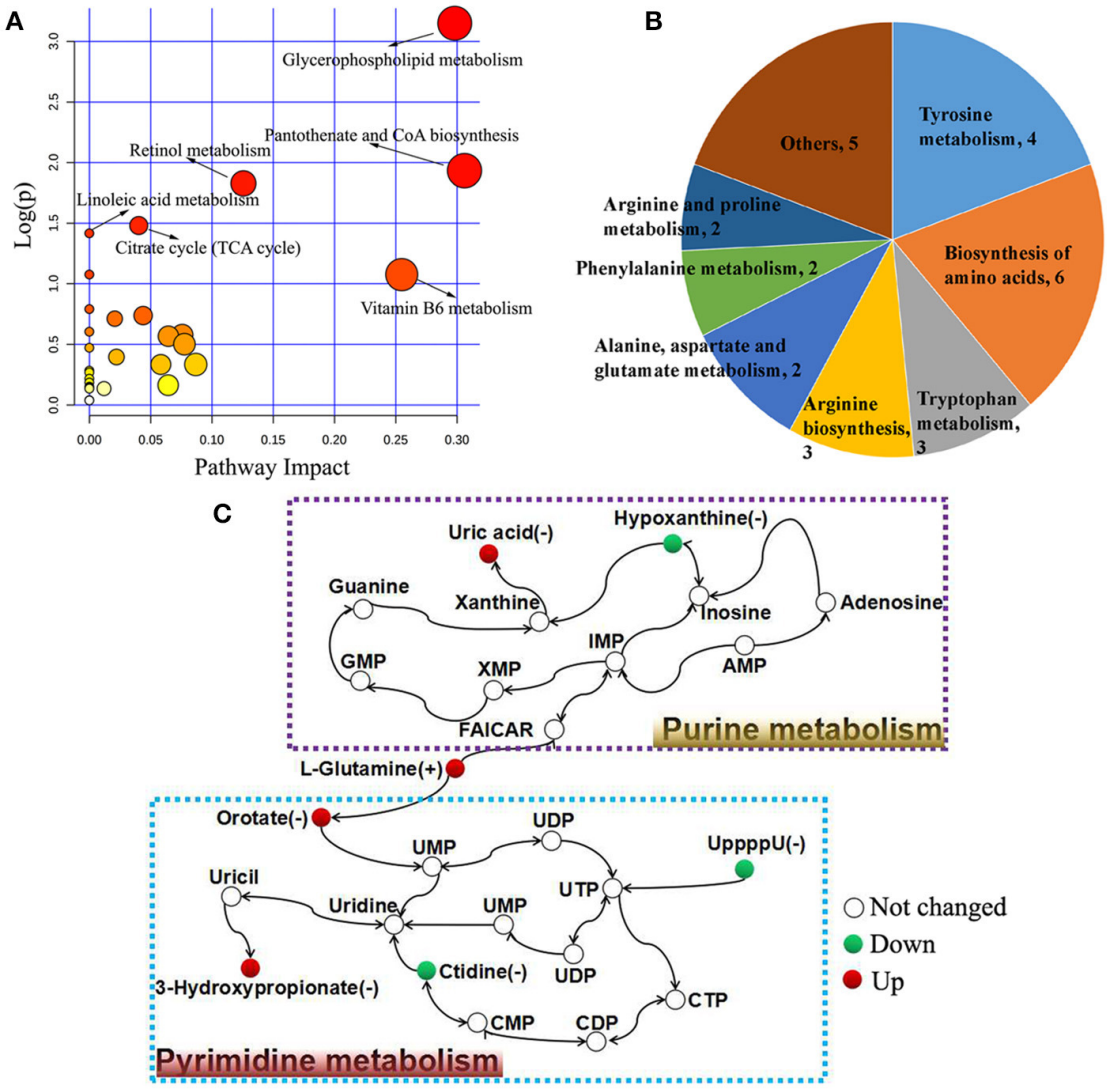
Pathway analysis of the identified differential metabolites between acutely infected and control mice in ESI+ mode and ESI− mode. **(A)** Pathway impact resulting from the differential metabolites using MetaboAnalyst 3.0. Small *p*-value and big pathway impact factor indicate that the pathway is greatly influenced. **(B)** Distribution of differential metabolites involved in amino acid metabolism. **(C)** Schematic overview of the differential metabolites involved in purine metabolism and pyrimidine metabolism. Red represents up-regulated metabolites, green represents down-regulated metabolites, and the open circles represent no change.

The biological pathways involved in the metabolism of the differentially expressed metabolites and their biological roles were determined by enrichment analysis using MetaboAnalyst. Six perturbed metabolic pathways showed lower *p*-values and higher pathway impact between acutely infected and control mice (Figure 5A). These included pathways related to metabolism of glycerophospholipid, pantotherate and CoA biosynthesis, retinol, linoleic acid, citrate cycle, and vitamin B6. Metabolism and biosynthesis of several amino acid were significantly perturbed during *T. gondii* acute infection (Figure 5B). Differential metabolites between acutely infected and control mice, involved in amino acid metabolism, were determined and detailed information is listed in Table S2. Interestingly, L-glutamine, a metabolite involved in mitochondrial metabolism, was up-regulated. Through the KEGG analysis, we found uric acid and hypoxanthine perturbed in purine metabolism, and orotate, 3-hydroxypropionate, ctidine, and UppppU were affected in pyrimidine metabolism (Figure 5C).

Among infection-related dysregulated pathways, “alanine, aspartate and glutamate metabolism,” “glycerophospholipid metabolism,” “terpenoid backbone biosynthesis,” and “retinol metabolism” were the top 4 perturbed metabolic pathways between acutely and chronically infected mice (Figure S7). Meanwhile, 13 metabolites, gentisic acid, imidazole acetol-phosphate, (R) 2,3-dihydroxy-3-methylvalerate, L-tyrosine, L-glutamine, L-arginine, citrulline, DL-dopa, 5-hydroxy-2-oxo-4-ureido-2,5-dihydro-1H-imidazole-5-carboxylate, L-cystathionine, indoleacetaldehyde, 5-hydroxykynurenamine and 3-methyl-1-hydroxybutyl-ThPP were altered and involved in amino acid metabolism (Table S3).

## Discussion

Infections by *T. gondii* are widely prevalent in human beings and animals world wide. It has been reported that the majority of human toxoplasmosis cases were associated with strains of a genotype II (Dardé et al., 1992; Howe and Sibley, 1995). In food animals, most of the *T. gondii* isolates thus far have been of type II or III (Literák et al., 1998; Mondragon et al., 1998; Owen and Trees, 1999). The oral route is the natural route for acquiring human and animal pathogen *T. gondii* and infection via the consumption of water contaminated with *T. gondii* oocysts has been frequently reported (Dubey, 2004; Hill et al., 2011). However, knowledge about the pathophysiology of oocyst-induced toxoplasmosis is still limited. Also, there is a pressing need for new biomarkers that can reliably detection infection and stratify patients infected with *T. gondii* oocysts. Metabolomics offers a powerful means to investigate host-parasite interactions at the biochemical level and to discover novel biomarkers of infection (Lakshmanan et al., 2012; Zhou et al., 2015, 2016b). In this study, a non-targeted LC-MS/MS-based method was used to explore different metabolic signatures of serum from mice during acute and chronic stage of oocyst-induced toxoplasmosis compared with uninfected controls and to identify metabolites and metabolic pathways that become dysregulated during *T. gondii* infection.

Clinical signs of infected mice were observed during the whole infection process. Mice at 11 dpi showed typical signs of acute infection, which was accompanied by the parasite distribution throughout the whole body except muscles. As infection progressed animals restored their health conditions at 33 dpi where the parasites were only detected in the brain, heart and muscle. These data indicate a correlation between the severity of toxoplasmosis and parasite distribution. Plots from the multivariate analyses (PCA and PLS-DA) clearly showed time-course biosignatures of mice metabolic response to *T. gondii* infection (Figure 1). As shown in Figure 2 and Figure S5, PLS-DA demonstrated clear separation among different mice groups. Comparison of the serum metabolic profiles of acutely infected and control mice showed marked differences in the levels of metabolites belonged to lipids, fatty acids, amino acids, products of amino acid metabolism, nucleotides, benzenoids, phenylpropanoids, and alkaloids. These results indicate that acute *T. gondii* infection can induce systemic metabolic perturbations in the metabolism of several chemical classes of macromolecules including traumatin, a product of α-linolenic acid (ALA) metabolism, not previously known to exist in *T. gondii*. In contrast, chronic infection did not seem to significantly alter the serum metabolic profile; only minor alterations were observed in fatty acyls, benzenes, steroids and glycerophospholipids. These differences are consistent with our previous metabolomics findings in mice infected with *T. gondii* cysts (Zhou et al., 2015). Thirty-five metabolites detected in ESI+ mode varied at acute and chronic infections. Except for traumatin, all of these differential metabolites showed decreased levels, which might be driven by the inappetence and decreased food intake of infected mice.

To ensure its survival and maximize parasite replication *T. gondii* modulates host cell environment, such as scavenging lipid precursors from host cell for growth and membrane biogenesis (Gupta et al., 2005; Bisanz et al., 2006). Lipids have various biological functions and are fundamental components of biological membranes and organelles. We detected alterations in fatty acyls and glycerophospholipids. Specifically, an increase in a number of intermediates involved in glycerophospholipids synthesis was noted and lysoPCs, lysoPEs, and PCs were the top 3 perturbed metabolites (Figure 4A). Also, we observed a reduction in the levels of the majority of lysophospholipids, especially during acute infection. Lysophospholipids are metabolite derivatives of membrane phospholipids via the action of phospholipase A_2_ (PLA_2_) enzyme and serve as precursors for inflammatory mediators (Libby et al., 2002). Hence, the reduced concentration of lysophospholipids is likely to be due to inhibition of PLA_2_, probably mediated by *T. gondii* as an evasion mechanism to reduce the host inflammatory response during acute infection. However, PLA_2_ is known to play a key role in *T. gondii* infection (Gomez-Marín et al., 2002). Whether *T. gondii* has a dual effect on PLA_2_, selectively inhibits the production of inflammatory mediators without inhibiting the beneficial properties of PLA_2_ to the parasite establishment, remains to be investigated.

Interestingly, one of the putatively annotated differentially abundant metabolites in our analysis belonged to a plant-like ALA metabolism pathway. This metabolite, annotated as traumatin (2-dodeceno-1-al-10-carboxylic acid), showed increased levels especially during the acute stage of infection. Traumatin has been also detected in *Plasmodium falciparum*-infected human plasma (Lakshmanan et al., 2012). Traumatin is the aldehyde derivative of the plant hormone Traumatic acid (TA, trans-2-dodecenedioic acid), and both are called wound hormones, because they appear in high amount around wounds and can stimulate cell proliferation in plants. TA plays an important role in the regulation of growth and metabolism in water plant, Wolffiaarrhiza Wimm, which was thought to maintain the redox balance to protect plant cells from oxidative stress (Pietryczuk and Czerpak, 2012). TA caused a reduction in membrane phospholipid peroxidation and exhibited protective antioxidative properties against reactive oxygen species (ROS) production as well as capacity to stimulate human skin fibroblasts proliferation (Jabłonska-Trypuć et al., 2016). The relative importance of traumatin in *T. gondii* infection is still unknown. Traumatin can potentially have a novel role in the interplay between *T. gondii* and host. Further investigation will be needed to determine the significance of changes in traumatin levels during *T. gondii* infection.

Biological amine-containing metabolites represent a large group of organic molecules including amino acids, other biogenic amines, and signaling molecules, such as neurotransmitters. They play essential roles in many biological functions. Their identification and quantification in biofluids provided significant insights into the pathogenesis of various diseases, such as cancer, diabetes and Parkinson's disease (Przedborski et al., 2000; Dronavalli et al., 2008; Wang et al., 2013). Our results revealed perturbations in 26 amine-containing metabolites involved in amino acid and nucleotide (purine and pyrimidine) metabolic pathways. L-Arginine, an amino acid mainly metabolized either by inducible nitric oxide synthases or by arginase 1 in myeloid cells was found decreased during *T. gondii* infection. *T. gondii* is an arginine auxotroph and arginine starvation was proven to efficiently trigger differentiation of replicative tachyzoites into bradyzoites contained within cyst-like structures (Fox et al., 2004). As shown in Table S2, the levels of 4 differential metabolites, L-tyrosine, 4-hydroxycinnamic acid, gentisic acid, and homogentisic acid, decreased. The increased serum level of the essential amino acid phenylalanine (Phe) together with elevated Phe to tyrosine (Tyr) ratio is also interesting. This finding is expected because inflammatory responses associated with *T. gondii* infection may interfere with Phe metabolism and diminishes the conversion of Phe to Tyr, probably via impairment of the phenylalanine-hydroxylase (PAH), rate-limiting in the biosynthesis of dopamine. Thus, our finding is in agreement with previous reports relating behavioral changes in mice or psychiatric problems in humans infected with *T. gondii* to effect of the parasite on the equilibrium of the neurotransmitter dopamine (Elsheikha and Zhu, 2016). Although we did not measure dopamine in our study a low level of dopamine can be implied from the measured high Phe/Tyr ratio. This reduced dopamine occurred during acute infection (11 dpi) is consistent with previous reports where 14% increase in brain dopamine levels was detected in mice upon establishment of chronic infection (Stibbs, 1985); a result which was supported by the increased level of serum dopamine in chronically infected mice (Zhou et al., 2015).

The level of Tryptophan (Trp), another essential amino acid, was found decreased in our study. This is biologically plausible because Trp depletion via IFN-mediated IDO (Indoleamine 2,3-dioxygenase, a key enzyme of Trp metabolism) induction has been proposed as anti-parasitic response to deprive *T. gondii* tachyzoites of this essential amino acid (Silva et al., 2002). Inhibition of IDO can result in the rejection of allogenic fetuses, which suggests tryptophan breakdown is necessary for maintaining aspects of immune tolerance (Moffett and Namboodiri, 2003). Trp also acts as a precursor for the synthesis of the neurotransmitters melatonin and serotonin and hence, any reduction in serum Trp levels will lead to a reduction in serotonin synthesis, which contributes to the occurrence of psychopathological disorders. Although immune-related IDO activation has been proposed as a defense mechanism for starving *T. gondii* of Trp and inhibiting its growth this hypothesis can be challenged because the upregulation of IDO activity and the depletion of Trp did not seem to inhibit the parasite growth and reproduction of malarial parasite in mice (Sanni et al., 1998).

Our results also showed interesting pathways associated with infection, such as alanine, aspartate and glutamate metabolism (Figure S7). This finding agrees with the previous observation that *T. gondii* infection of pigs, significantly influences the plasma levels of alanine aminotransferase (ALT) 21 and 32 days after infection with tachyzoites of RH strain (Miranda et al., 2015). L-glutamine, as an important carbon source when glucose uptake is inhibited during *T.gondii* infection, was found upregulated in the acute infection stage. Glutamine was catabolized via the oxidative tricarboxylic acid (TCA) cycle and generates γ-aminobutyric acid and additional molecules that are essential for the intracellular growth of *T. gondii* (MacRae et al., 2012). Also, CCR5-deficient (CCR5^−/−^) mice infected with *T. gondii* exhibited increased level of ALT 8 dpi compared to uninfected mice (Bonfá et al., 2014). Additionally, treatment with 6-trifluoromethyl-2-thiouracil (KH-0562) or pyrimethamine was found to restore the elevated level of ALT in infected mice to the normal level, which was thought to be due to the anti-*T. gondii* activity and subsequent inhibition of hepatotoxicity derived from *T. gondii* infection (Choi et al., 2014). Hypoxanthine and other purines are essential nutrients that the intracellular *T. gondii* parasite salvages from the surrogate host cell to sustain its growth and proliferation. Hypoxanthine was found down-regulated, indicating that this compound is rapidly consumed in purine biosynthesis during acute infection. Four pyrimidine metabolites were identified: two upregulated (orotate and 3-hydroxypropionate) and two downregulated (ctidine and UpppU).

In conclusion, this study has demonstrated the ability of LC-MS/MS-based metabolomics to detect a broad range of differential metabolites in mice serum and to detected serum metabolic signatures that strongly distinguished between acutely and chronically infected mice. Our approach has enabled us to confirm some of the known metabolites, as well as unravel a hitherto undiscovered aspect of *T. gondii* pathobiology. We revealed differential metabolites related to alterations in metabolism of lipids (e.g., lysophospholipids) and amino acid (phenylalanine, tyrosine, tryptophan, alanine, aspartate, and glutamate). We also identified several dysregulated metabolic pathways involved in the pathogenesis of *T. gondii* infection including for the first time the existence of a plant-like traumatin molecule, belonging to α-linolenic acid (ALA) pathway, known to exist in plants but not known to exist in *T. gondii* until now. Further studies will be required to more completely dissect the relevance of the amine-containing metabolites identified in this study and to investigate the presence of additional intermediates of the plant ALA pathway in *T. gondii*–infected samples. It is hoped that our findings add to the growing body of literature that describes the application of “omics” methods to better understand *T. gondii* biology and address relevant biological and clinically important questions. Data generated from these undertakings should foster our efforts toward discovering new disease biomarkers and effective anti-*T. gondii* therapeutics.

## Data availability statement

The metabolomics data are available in the MetaboLights database [MTBLS418].

## Author contributions

X-QZ and C-XZ conceived and designed the experiments. C-XZ, WC, and X-QC performed the experiments. C-XZ, HE and S-YH contributed reagents, materials, analysis tools. C-XZ analyzed the data and wrote the paper. HE and X-QZ critically revised the manuscript. All authors read and approved the final version of the manuscript.

### Conflict of interest statement

The authors declare that the research was conducted in the absence of any commercial or financial relationships that could be construed as a potential conflict of interest.

## Supplementary material

Figure S1 Representative image of purified sporulated *Toxoplasma gondii* oocysts used in the study.

Figure S2 *Toxoplasma gondii* cyst recovered from homogenized mouse brain tissue at 33 DPI.

Figure S3 Histopathological changes in the splenic tissues from *Toxoplasma gondii*-infected mice. Photomicrographs of splenic tissue sections stained with hematoxylin and eosin (original magnification, × 100). **(A)** Spleen H&E section from a mouse on day 11 postinfection. Spleen shows a loss and fusion of the germinal centers in the white pulp and undefined boundary between the white pulp and the red pulp. **(B)** On day 33 postinfection, spleen shows mild hyperplasia and some lymphoid follicles tended to coalesce in the white pulp, whereas the red pulp was non-reactive. **(C)** Spleen section from a healthy, uninfected mouse. Spleen shows a normal histology with a clear structure of red (R) and white (W) pulps and normal cell density.

Figure S4 Principle component analysis (PCA) scores plots of serum samples obtained from *Toxoplasma gondii*-infected mice compared to quality control (QC) samples. **(A)** PCA scores plot for metabolites obtained in **(A)** positive ion mode (ESI+) and **(B)** negative ion mode (ESI−). Blue dots indicate QC samples and green dots indicate tested samples.

Figure S5 Discrimination between acutely infected, chronically infected and control mice based on ESI− mode-derived metabolic phenotype of serum. **(A–C)** Partial least squares-discriminate analysis (PLS-DA) score plots of **(A)** acutely infected vs. control, **(B)** chronically infected vs. control and **(C)** acutely infected vs. chronically infected. **(D–E)** Heat maps of the top differential metabolites for **(D)** acutely infected vs. control and **(E)** acutely infection vs. chronically infected. *Abbreviations:* AI, acutely infected; CI, chronically infected; Con, control.

Figure S6 Top 15 enriched metabolite classes identified between the chronically infected and control mice in ESI+ mode. Red and green color indicate up- and down-regulated metabolites, respectively. The bars on x-axis represent the number of metabolites for the chemical classes mentioned on the y-axis.

Figure S7 Summary of aberrant pathways of the significantly altered metabolites between acutely infected and chronically infected mice as analyzed by MetaboAnalyst. Plot shows the matched pathways depicted according to *p*-value from pathway enrichment analysis and pathway impact score from pathway topology analysis. Color gradient and circle size indicate the significance of the pathway ranked by *p*-value (yellow: higher *p*-values and red: lower *p*-values) and pathway impact score (the larger the circle the higher the impact score), respectively. Significantly affected pathways with low *p*-value and high pathway impact score are identified by name.

Table S1 Detection of *T. gondii* by PCR amplification.

Table S2 List of dysregulated metabolites involved in amino acid metabolism between acutely infected and control groups.

Table S3 List of dysregulated metabolites involved in amino acid metabolism between acutely infected and chronically infected groups.
