# Supplementary figures and images for: Serum Metabolic Profiling of Oocyst-Induced Toxoplasma gondii Acute and Chronic Infections in Mice Using Mass-Spectrometry
Source: Front Microbiol. 2018 Jan 4;8:2612. doi: 10.3389/fmicb.2017.02612 (PMC5761440; doi:10.3389/fmicb.2017.02612)

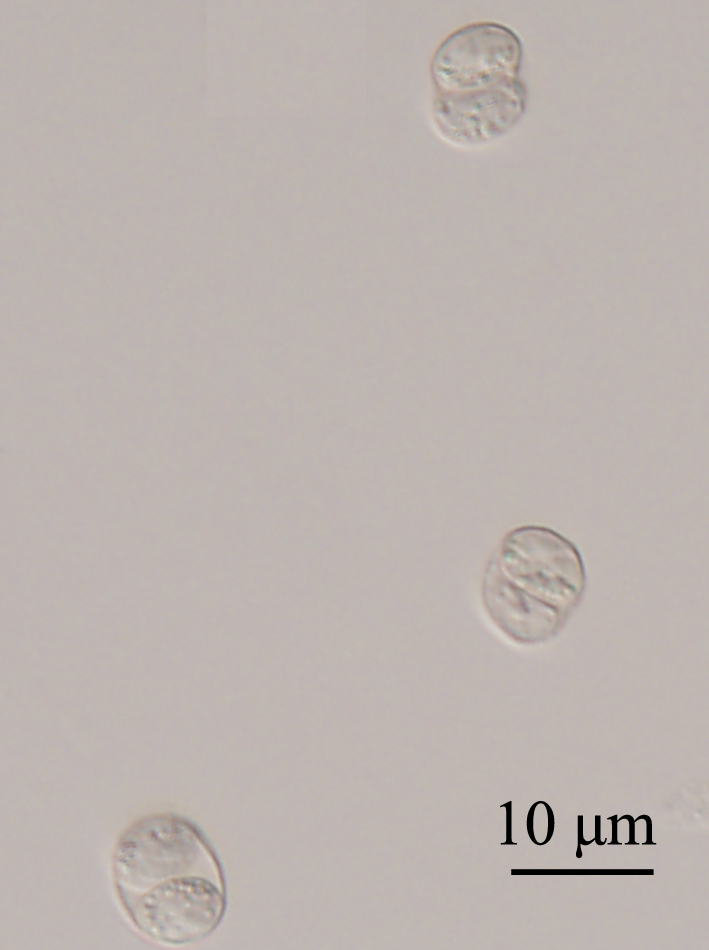

Supplement: Figure S1 — Representative image of purified sporulated Toxoplasma gondii oocysts used in the study. [file FigureS1.TIF]

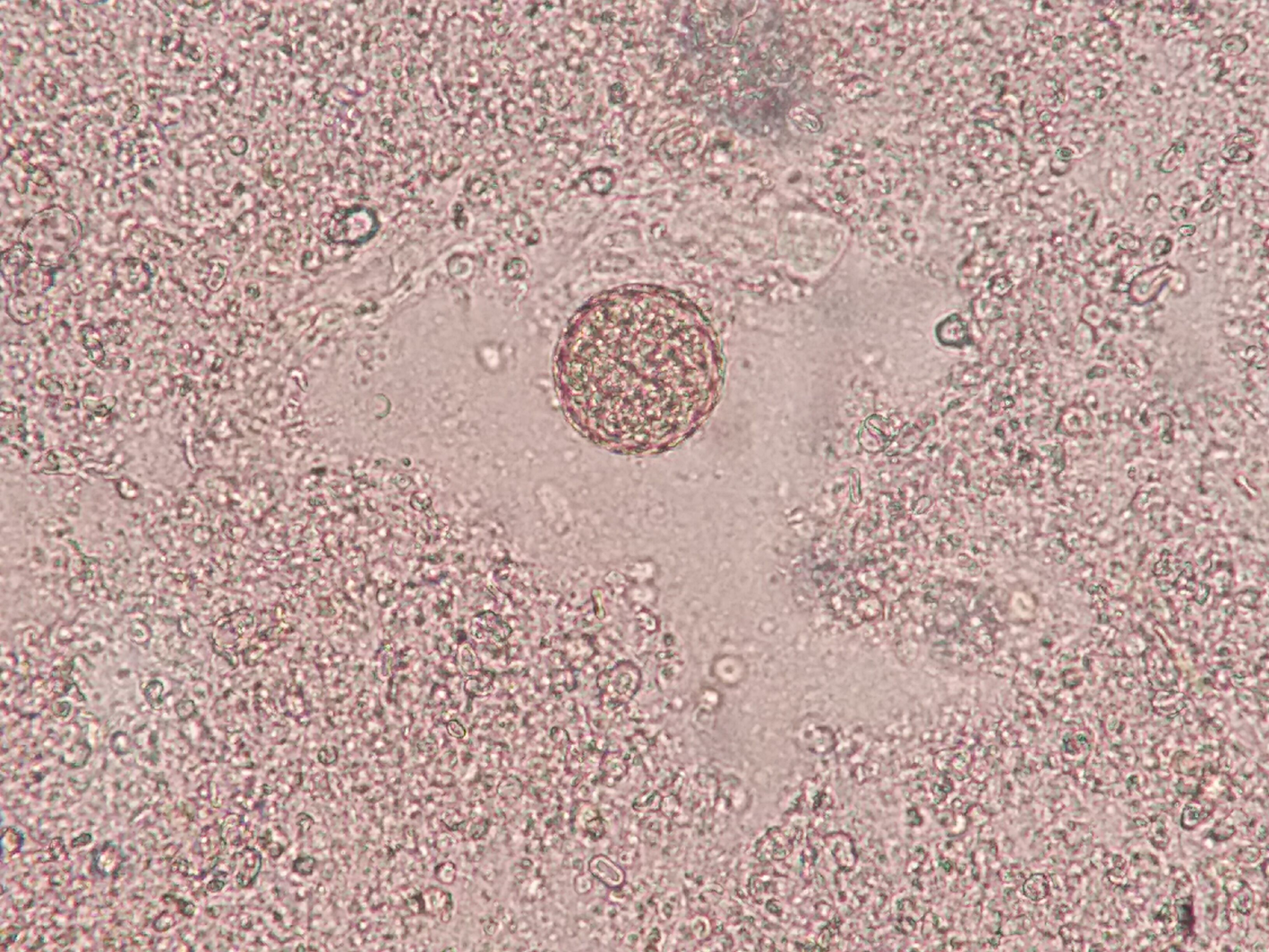

Supplement: Figure S2 — Toxoplasma gondii cyst recovered from homogenized mouse brain tissue at 33 DPI. [file FigureS2.TIF]

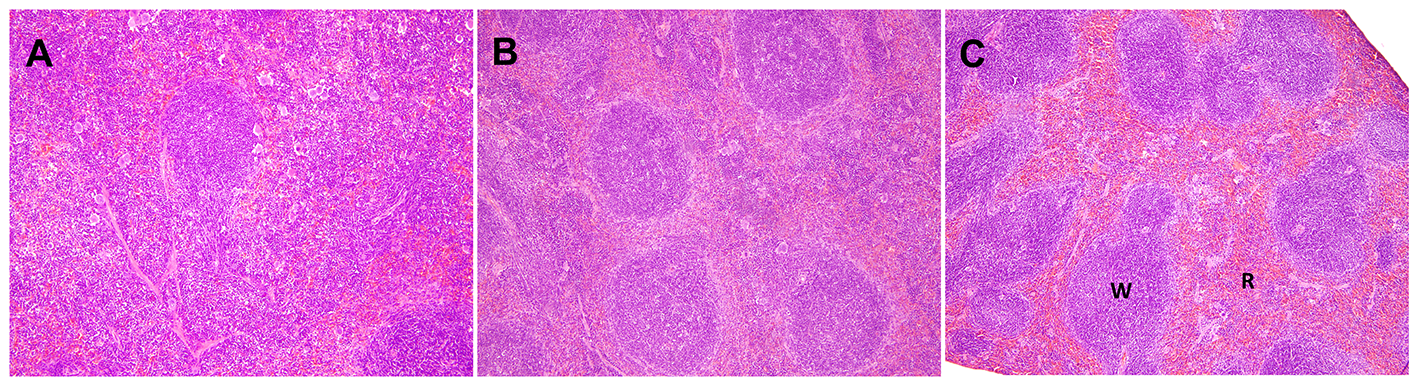

Supplement: Figure S3 — Histopathological changes in the splenic tissues from Toxoplasma gondii-infected mice. Photomicrographs of splenic tissue sections stained with hematoxylin and eosin (original magnification, × 100). (A) Spleen H&E section from a mouse on day 11 postinfection. Spleen shows a loss and fusion of the germinal centers in the white pulp and undefined boundary between the white pulp and the red pulp. (B) On day 33 postinfection, spleen shows mild hyperplasia and some lymphoid follicles tended to coalesce in the white pulp, whereas the red pulp was non-reactive. (C) Spleen section from a healthy, uninfected mouse. Spleen shows a normal histology with a clear structure of red (R) and white (W) pulps and normal cell density. [file FigureS3.tif]

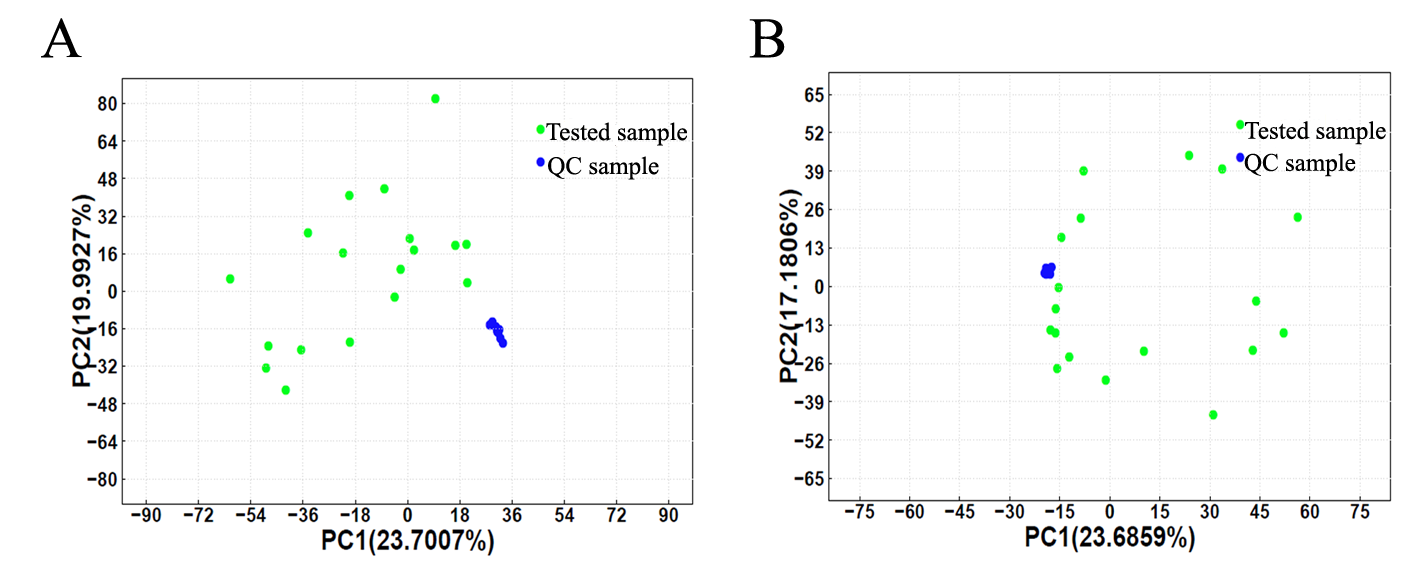

Supplement: Figure S4 — Principle component analysis (PCA) scores plots of serum samples obtained from Toxoplasma gondii-infected mice compared to quality control (QC) samples. (A) PCA scores plot for metabolites obtained in (A) positive ion mode (ESI+) and (B) negative ion mode (ESI−). Blue dots indicate QC samples and green dots indicate tested samples. [file FigureS4.TIF]

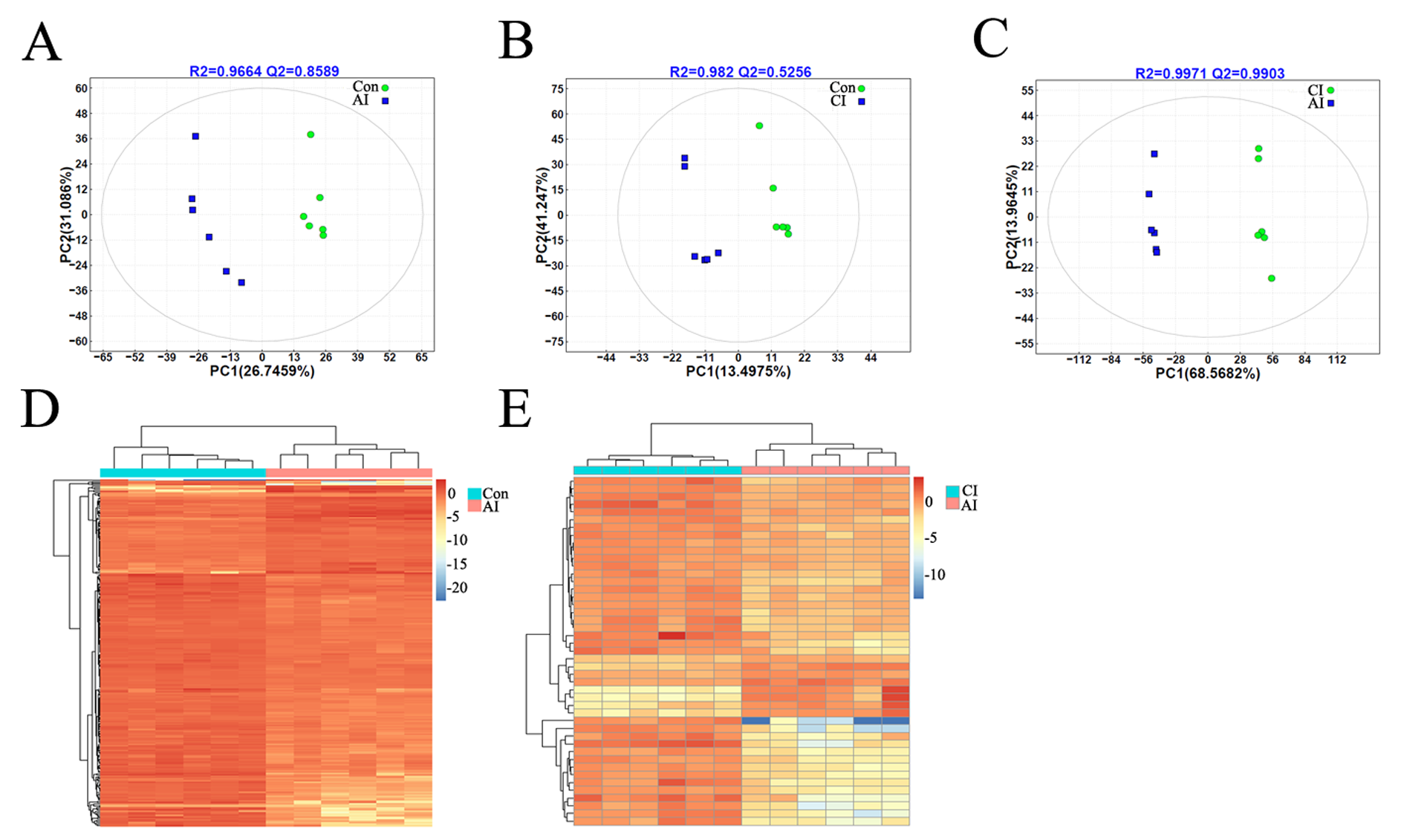

Supplement: Figure S5 — Discrimination between acutely infected, chronically infected and control mice based on ESI− mode-derived metabolic phenotype of serum. (A–C) Partial least squares-discriminate analysis (PLS-DA) score plots of (A) acutely infected vs. control, (B) chronically infected vs. control and (C) acutely infected vs. chronically infected. (D–E) Heat maps of the top differential metabolites for (D) acutely infected vs. control and (E) acutely infection vs. chronically infected. Abbreviations: AI, acutely infected; CI, chronically infected; Con, control. [file FigureS5.TIF]

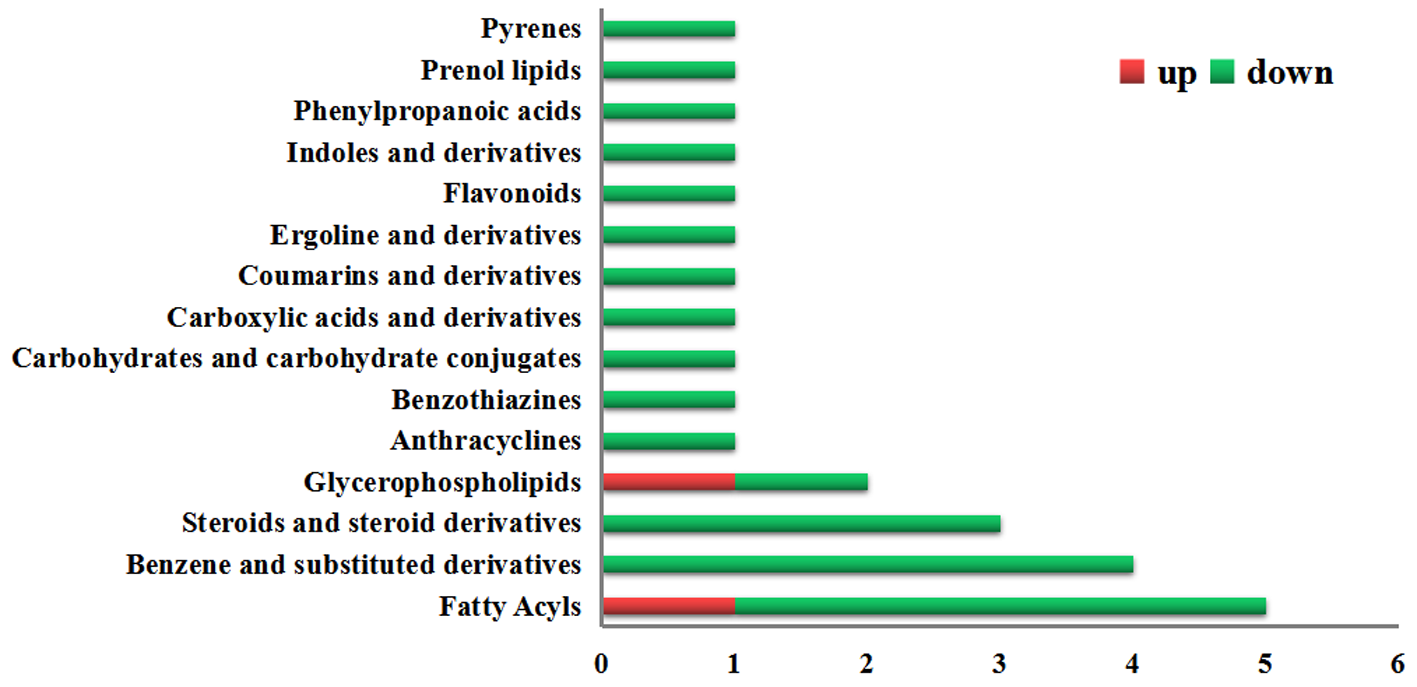

Supplement: Figure S6 — Top 15 enriched metabolite classes identified between the chronically infected and control mice in ESI+ mode. Red and green color indicate up- and down-regulated metabolites, respectively. The bars on x-axis represent the number of metabolites for the chemical classes mentioned on the y-axis. [file FigureS6.TIF]

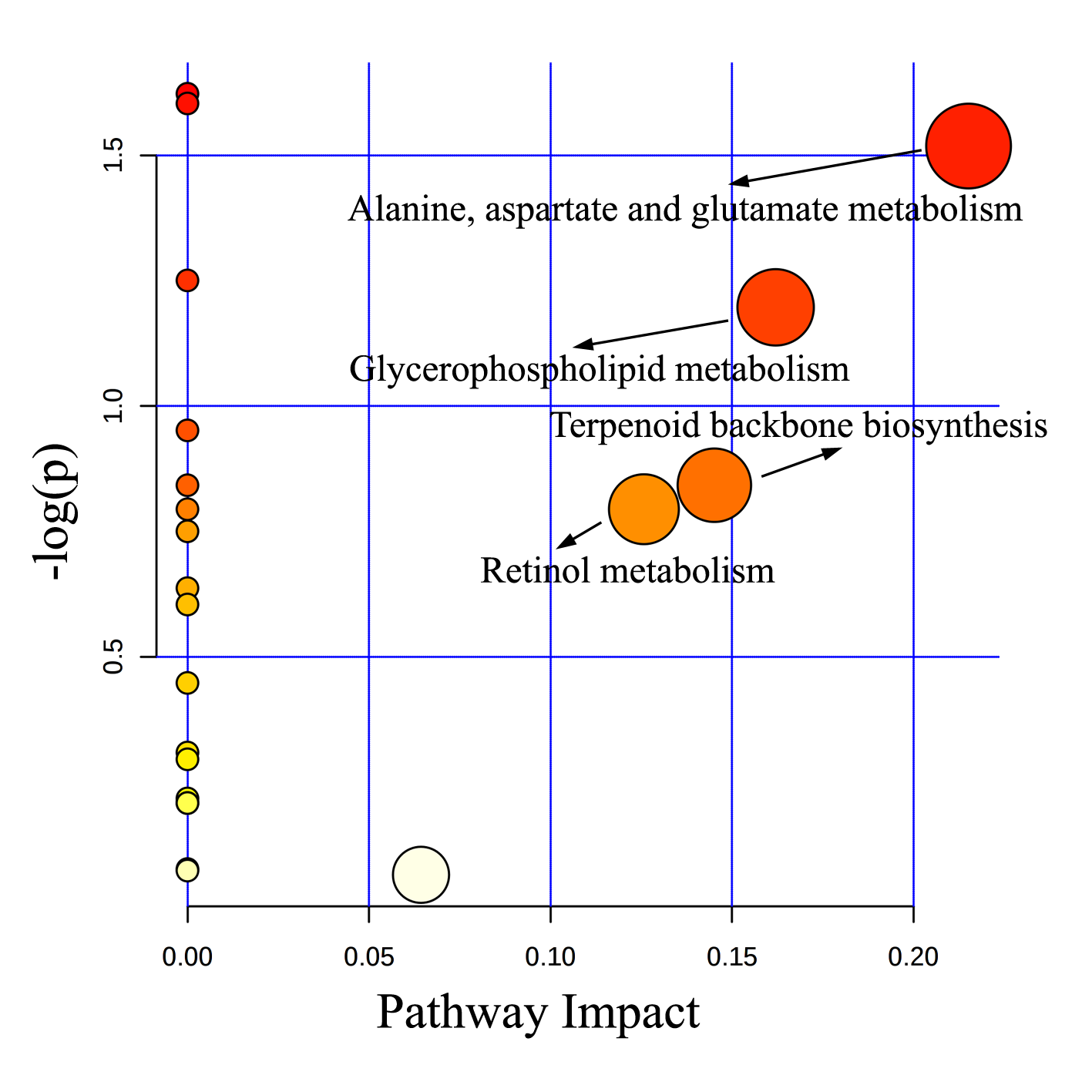

Supplement: Figure S7 — Summary of aberrant pathways of the significantly altered metabolites between acutely infected and chronically infected mice as analyzed by MetaboAnalyst. Plot shows the matched pathways depicted according to p-value from pathway enrichment analysis and pathway impact score from pathway topology analysis. Color gradient and circle size indicate the significance of the pathway ranked by p-value (yellow: higher p-values and red: lower p-values) and pathway impact score (the larger the circle the higher the impact score), respectively. Significantly affected pathways with low p-value and high pathway impact score are identified by name. [file FigureS7.TIF]
