# Supplementary material for: Serum Metabolic Profiling of Oocyst-Induced Toxoplasma gondii Acute and Chronic Infections in Mice Using Mass-Spectrometry
Source: Front Microbiol. 2018 Jan 4;8:2612. doi: 10.3389/fmicb.2017.02612 (PMC5761440; doi:10.3389/fmicb.2017.02612)
Supplement: Table S1 — Detection of T. gondii by PCR amplification. [file TableS1.DOC]

**Table S1 |** **Detection of *T. gondii* by PCR amplification.**

| Tissue | AI | CI | Con |
| --- | --- | --- | --- |
| Blood | ＋ | － | － |
| Brain | ＋ | ＋ | － |
| Heart | ＋ | ＋ | － |
| Kidney | ＋ | － | － |
| liver | ＋ | － | － |
| Lung | ＋ | － | － |
| Muscle | － | ＋ | － |
| Spleen | ＋ | － | － |
| Small intestine | ＋ | － | － |

＋indicates PCR positive;－indicates PCR negative; AI, CI, and Con denote acutely infected group, chronically infected group, and control group.
