# Supplementary material for: Serum Metabolic Profiling of Oocyst-Induced Toxoplasma gondii Acute and Chronic Infections in Mice Using Mass-Spectrometry
Source: Front Microbiol. 2018 Jan 4;8:2612. doi: 10.3389/fmicb.2017.02612 (PMC5761440; doi:10.3389/fmicb.2017.02612)
Supplement: Table S2 — List of dysregulated metabolites involved in amino acid metabolism between acutely infected and control groups. [file TableS2.DOC]

**Table S2 | List of dysregulated metabolites involved in amino acid metabolism between acutely infected and control groups.**

| **Mode** | **MS (m/z)** | **RT (min)** | **Metabolites (HMDB ID)** | **VIP** | **FC** | ***q*-value** | **CV** | **Metabolic pathways** |
| --- | --- | --- | --- | --- | --- | --- | --- | --- |
| ESI+ | 146.0612569 | 3.8551 | L-Tyrosine (HMDB00158) | 2.67 | 0.116 | 1.35E03 | ↓ | Tyrosine metabolism; Phenylalanine, tyrosine and tryptophan biosynthesis; Phenylalanine metabolism |
| ESI+ | 221.0199166 | 0.604766667 | 4-Hydroxycinnamic acid (HMDB00755) | 1.83 | 0.346 | 1.93E03 | ↓ | Tyrosine metabolism;  Phenylalanine metabolism |
| ESI- | 153.0180716 | 4.2474 | Gentisic acid (HMDB00152) | 2.27 | 0.291 | 4.76E02 | ↓ | Tyrosine metabolism |
| ESI- | 203.0130047 | 0.811333333 | Homogentisic acid (HMDB00130) | 1.01 | 0.758 | 1.65E02 | ↓ | Tyrosine metabolism |
| ESI+ | 198.0860826 | 0.604766667 | Citrulline (HMDB00904) | 2.01 | 0.261 | 1.80E02 | ↓ | Biosynthesis of amino acids;  Arginine biosynthesis |
| ESI+ | 175.1200396 | 0.5762 | L-Arginine (HMDB00517) | 1.81 | 0.323 | 1.35E02 | ↓ | Biosynthesis of amino acids; Arginine biosynthesis; D-Arginine and D-ornithine metabolism |
| ESI+ | 169.0595533 | 0.590483333 | L-Glutamine (HMDB00641) | 1.15 | 1.670 | 1.06E02 | ↑ | Biosynthesis of amino acids;  Arginine biosynthesis;  Alanine, aspartate and glutamate metabolism;  D-Glutamine and D-glutamate metabolism |
| ESI- | 203.0811471 | 3.9838 | L-Tryptophan (HMDB00929) | 2.63 | 0.192 | 3.59E03 | ↓ | Biosynthesis of amino acids; Tryptophan metabolism; Glycine, serine and threonine metabolism |
| ESI- | 164.0704063 | 3.477316667 | L-Phenylalanine (HMDB00159) | 1.02 | 1.311 | 4.49E03 | ↑ | Biosynthesis of amino acids |
| ESI+ | 319.1459315 | 9.622116667 | Indoleacetaldehyde (HMDB01190) | 1.14 | 0.609 | 9.14E03 | ↓ | Tryptophan metabolism |
| ESI+ | 383.1706457 | 11.21941667 | 5-Hydroxykynurenamine (HMDB04076) | 1.92 | 3.570 | 4.42E02 | ↑ | Tryptophan metabolism |
| ESI+ | 541.0788368 | 8.339133333 | Glucosamine 6-phosphate (HMDB01254) | 1.09 | 0.614 | 2.31E02 | ↓ | Alanine, aspartate and glutamate metabolism |
| ESI- | 146.0447717 | 0.63335 | L-4-Hydroxyglutamate semialdehyde (HMDB06556) | 1.64 | 0.500 | 6.21E03 | ↓ | Arginine and proline metabolism |
| ESI+ | 529.1525251 | 8.7456 | 3-Methyl-1-hydroxybutyl-ThPP (HMDB06865) | 2.99 | 11.9319 | 7.55E03 | ↑ | Valine, leucine and isoleucine degradation |

RT, retention time; VIP, variable importance for projection; CV, Content variance; AI, CI, and Con denote acutely infected group, chronically infected group, and control group; FC, Fold change; *q*-value, adjusted *p* value calculated by the two-tailed Wilcoxon rank-sum tests after false discovery rate correction.
