# Supplementary material for: Serum Metabolic Profiling of Oocyst-Induced Toxoplasma gondii Acute and Chronic Infections in Mice Using Mass-Spectrometry
Source: Front Microbiol. 2018 Jan 4;8:2612. doi: 10.3389/fmicb.2017.02612 (PMC5761440; doi:10.3389/fmicb.2017.02612)
Supplement: Table S3 — List of dysregulated metabolites involved in amino acid metabolism between acutely infected and chronically infected groups. [file TableS3.DOC]

Table S3|List of dysregulated metabolites involved in amino acid metabolism between acutely infected and chronically infected groups.

| **Mode** | **MS (m/z)** | **RT (min)** | **Metabolites (HMDB ID)** | **VIP** | **FC** | ***q*-value** | **CV** | **Metabolic pathways** |
| --- | --- | --- | --- | --- | --- | --- | --- | --- |
| ESI+ | 146.0612569 | 3.8551 | L-Tyrosine (HMDB00158) | 2.66 | 0.123 | 1.13E03 | ↓ | Tyrosine metabolism; Phenylalanine, tyrosine and tryptophan biosynthesis; Phenylalanine metabolism |
| ESI- | 254.9937508 | 4.433183333 | Imidazole acetol-phosphate (HMDB12236) | 1.80 | 6.590 | 1.88E02 | ↑ | Histidine metabolism; Biosynthesis of amino acids |
| ESI- | 153.0180716 | 4.2474 | Gentisic acid (HMDB00152) | 2.27 | 0.160 | 3.27E02 | ↓ | Tyrosine metabolism |
| ESI- | 183.0433479 | 5.79025 | (R) 2,3-Dihydroxy-3-methylvalerate (HMDB12140) | 1.46 | 0.269 | 7.55E03 | ↓ | Valine, leucine and isoleucine biosynthesis; Biosynthesis of amino acids |
| ESI+ | 198.0860826 | 0.604766667 | Citrulline (HMDB00904) | 2.11 | 0.274 | 2.15E02 | ↓ | Biosynthesis of amino acids;  Arginine biosynthesis |
| ESI+ | 175.1200396 | 0.5762 | L-Arginine (HMDB00517) | 1.58 | 0.438 | 3.59E02 | ↓ | Biosynthesis of amino acids; Arginine biosynthesis; D-Arginine and D-ornithine metabolism |
| ESI+ | 169.0595533 | 0.590483333 | L-Glutamine (HMDB00641) | 1.20 | 1.629 | 3.62E02 | ↑ | Biosynthesis of amino acids;  Arginine biosynthesis;  Alanine, aspartate and glutamate metabolism;  D-Glutamine and D-glutamate metabolism |
| ESI+ | 319.1459315 | 9.622116667 | Indoleacetaldehyde (HMDB01190) | 1.22 | 0.623 | 1.06E03 | ↓ | Tryptophan metabolism |
| ESI+ | 383.1706457 | 11.21941667 | 5-Hydroxykynurenamine (HMDB04076) | 1.94 | 3.207 | 3.33E02 |  | Tryptophan metabolism |
| ESI+ | 529.1525251 | 8.7456 | 3-Methyl-1-hydroxybutyl-ThPP (HMDB06865) | 2.65 | 8.502 | 4.37E03 | ↑ | Valine, leucine and isoleucine degradation |
| ESI+ | 215.1015478 | 0.5762 | DL-Dopa (HMDB00609) | 1.06 | 0.274 | 2.15E02 | ↓ | Tyrosine metabolism |
| ESI+ | 240.1007633 | 9.358516667 | L-Cystathionine (HMDB00099) | 1.07 | 1.48 | 1.41E02 | ↑ | Glycine, serine and threonine metabolism; Cysteine and methionine metabolism; Biosynthesis of amino acids |

RT, retention time; VIP, variable importance for projection; CV, Content variance; AI, CI, and Con denote acutely infected group, chronically infected group, and control group; FC, Fold change; *q*-value, adjusted *p* value calculated by the two-tailed Wilcoxon rank-sum tests after false discovery rate correction.
